# Factors associated with health facility deliveries among mothers living in hospital catchment areas in Rukungiri and Kanungu districts, Uganda

**DOI:** 10.1186/s12884-021-03789-3

**Published:** 2021-04-26

**Authors:** Richard K. Mugambe, Habib Yakubu, Solomon T. Wafula, Tonny Ssekamatte, Simon Kasasa, John Bosco Isunju, Abdullah Ali Halage, Jimmy Osuret, Constance Bwire, John C. Ssempebwa, Yuke Wang, Joanne A. McGriff, Christine L. Moe

**Affiliations:** 1 Department of Disease Control and Environmental Health, School of Public Health, Kampala, College of Health Sciences, Makerere University, P.O Box 7072, Kampala, Uganda; 2 The Center for Global Safe Water, Sanitation and Hygiene, Rollins School of Public Health, Emory University, 1518 Clifton Rd. NE, Atlanta, GA 30322 USA; 3 Department of Epidemiology and Biostatistics, School of Public Health, Kampala, College of Health Sciences, Makerere University, P.O Box 7072, Kampala, Uganda

**Keywords:** Childbirth, Health facility delivery, Private healthcare facilities, Public healthcare facilities, Mothers, Uganda

## Abstract

**Background:**

Health facility deliveries are generally associated with improved maternal and child health outcomes. However, in Uganda, little is known about factors that influence use of health facilities for delivery especially in rural areas. In this study, we assessed the factors associated with health facility deliveries among mothers living within the catchment areas of major health facilities in Rukungiri and Kanungu districts, Uganda.

**Methods:**

Cross-sectional data were collected from 894 randomly-sampled mothers within the catchment of two private hospitals in Rukungiri and Kanungu districts. Data were collected on the place of delivery for the most recent child, mothers’ sociodemographic and economic characteristics, and health facility water, sanitation and hygiene (WASH) status. Modified Poisson regression was used to estimate prevalence ratios (PRs) for the determinants of health facility deliveries as well as factors associated with private versus public utilization of health facilities for childbirth.

**Results:**

The majority of mothers (90.2%, 806/894) delivered in health facilities. Non-facility deliveries were attributed to faster progression of labour (77.3%, 68/88), lack of transport (31.8%, 28/88), and high cost of hospital delivery (12.5%, 11/88). Being a business-woman [APR = 1.06, 95% CI (1.01–1.11)] and belonging to the highest wealth quintile [APR = 1.09, 95% CI (1.02–1.17)] favoured facility delivery while a higher parity of 3–4 [APR = 0.93, 95% CI (0.88–0.99)] was inversely associated with health facility delivery as compared to parity of 1–2. Factors associated with delivery in a private facility compared to a public facility included availability of highly skilled health workers [APR = 1.15, 95% CI (1.05–1.26)], perceived higher quality of WASH services [APR = 1.11, 95% CI (1.04–1.17)], cost of the delivery [APR = 0.85, 95% CI (0.78–0.92)], and availability of caesarean services [APR = 1.13, 95% CI (1.08–1.19)].

**Conclusion:**

Health facility delivery service utilization was high, and associated with engaging in business, belonging to wealthiest quintile and having higher parity. Factors associated with delivery in private facilities included health facility WASH status, cost of services, and availability of skilled workforce and caesarean services.

**Supplementary Information:**

The online version contains supplementary material available at 10.1186/s12884-021-03789-3.

## Background

Maternal mortality remains a leading cause of death among females aged 15–49 years particularly in resource-poor countries [1, 2], where 99% of global maternal deaths occur [3]. Despite various global initiatives focused on maternal health, in 2015 alone, an estimated 830 women died daily as a result of complications related to pregnancy and childbirth, and 550 of those deaths occurred in sub-Saharan Africa [3]. Maternal mortality is largely related to poor access to obstetric care especially during the intrapartum period [4, 5]. Although it has been widely acknowledged that hospital deliveries provide an effective opportunity for minimizing maternal and neonatal deaths [6–8], a significant fraction of mothers in low-income countries continues to deliver without the assistance of skilled health workers [9–11]. In these countries, most pregnant women deliver under the care of either their family members, traditional birth attendants, or no one at all [12]. Efforts to reduce maternal and neonatal mortality include initiatives to increase the proportion of mothers delivering babies in health facilities, attended to by skilled health workers [13].

The choice of place to deliver a child is thus an important aspect for the health of the mother and baby. Decisions about where to give birth are influenced by social, cultural, and environmental factors [14, 15]. These factors can range from distance to a health facility, cost and quality of services [16, 17], and governance of the health facility [18]. Concerns related to efficiency, availability of services, and physical infrastructure in public facilities have been reported in some low- and middle-income countries [18] and may affect whether mothers deliver in health facilities or not. Adequate water, sanitation, and hygiene (WASH) can also be an important component in care-seeking decisions and the provision of basic health services [19]. Even when health facilities offer quality services, lack of access to adequate WASH services in facilities may discourage women from delivering in these facilities or cause delays in care-seeking [20].

According to the latest Uganda Demographic and Health Survey (UDHS), Uganda has a maternal mortality ratio of 336 deaths per 100,000 live births, which is among the highest in Eastern Africa, and about 27% of pregnant women in Uganda still do not deliver in health facilities [21]. Despite government interventions, such as the construction of more health facilities, training of community health workers to promote community health education, and linkage to facilities for pregnant women, facilities are still underutilized or inaccessible [22, 23]. Increasing health facility deliveries requires that women have the physical and financial means to access the facilities whilst being assisted to make decisions to seek these services [24, 25]. Most of the studies on determinants of child delivery location conducted in Uganda were not performed in remote areas where it is often difficult for mothers to travel to local health facilities [22, 26, 27]. Recognizing the paucity of information on the process and determinants of choice on childbirth location in hard to reach areas such as rural Western Uganda, this study aimed to explore the factors associated with health facility births, and draw comparisons between private and public health facilities, in order to inform the design of better approaches to increase childbirth in health facilities.

## Methods

### Study design and study setting

This was a cross-sectional cluster survey conducted within the catchment communities of two major private-not-for-profit facilities; Kisiizi hospital in Rukungiri District, and Bwindi Community Hospital in Kanungu District in Western Uganda. However, these communities were also served by some public health facilities at different levels. A catchment was defined as communities (sub-counties) which were reported to be having the majority of the clients for the two major private health facilities. In Uganda, the health care system is organised into a four-tier system (i.e., hospitals, health centres of levels IV, III, and II). Hospitals are considered higher-level facilities because they have consultant physicians and offer more specialized services, while health centres II, III, and IV, found at parish, sub-county, and county levels, respectively, are considered lower level facilities (or primary health care). These primary health care facilities offer basic services. For example, health center IIs offer outpatients consultations, while health center IIIs offer some inpatient care and some laboratory services. In addition to all the services provided at health centers II and III, health centers at level IV offer caesarean deliveries and blood transfusion services.

Kanungu district has an area of 1291.1 Km^2^ and an estimated population of 252,144 people and a population growth rate of 2.1% per annum [28]. Kanungu has two hospitals: one public hospital (Kambuga Hospital) in Kambuga Town council and one private hospital (Bwindi Community Hospital) on the outskirts of Bwindi impenetrable forest. Additionally, there are two health centre (HC) IVs, 10 HC IIIs, and 17 HC IIs. Rukungiri district covers an area of 1566.8 Km^2^ and had an estimated population of 314,694 people in 2016, with an annual growth rate of 3.0% [28]. Some of the major hospitals in Rukungiri district include Nyakibale hospital (public) and Kisiizi hospital (private). Rukungiri has 9 HC IVs, 9 HC IIIs, and 43 HC IIs.

### Study population

All women of reproductive age who had given birth within the last 12 months preceding the survey were considered. The inclusion and exclusion criteria are shown in Table 1.

**Table 1:** Inclusion and exclusion criteria

Inclusion criteria	Exclusion criteria
• Mothers of 18 and above years • Having a child of 0–12 months old • Minimum of at least 1 year of stay in the community. • Signed (or thumb-printed) informed consent document	• Mothers below 18 years of age. • Mothers with children reported to be above 12 months of age. • Visiting mothers and those who had stayed in the community for less than 1 year. • Refusal to provide consent for participation in the study. • Critically sick mothers

### Sample size determination and sampling procedures

#### Sample size estimation

To obtain the sample size, we used the Kish leslie formula for cross-sectional studies, with the following assumptions; an alpha of 0.05, power (1-beta) of 0.80, a sampling error of 5%, a nonresponse rate of 10%, and a statistically conservative 50% proportion of mothers that utilize health facility delivery services. This was done since there was limited data about hospital delivery utilization in rural areas in Uganda. We also considered a design effect of 2.0 to cater for potential differential clustering by catchment in the two districts. A sample of size of 846 was obtained and was rounded to make 900 participants.

This was a cross-sectional cluster survey where a sample was selected from Kisiizi and Bwindi health facility catchment areas. For this study, a cluster was defined as a village, which is the local administrative unit in Uganda. A total of 60 clusters, each consisting of 15 households, were randomly selected from the two catchment areas (major sub-counties served by each of the two major hospitals) leading to a minimum target sample size of 900 households.

#### Selection of clusters and households

We used a two-stage sampling approach [29]. First, we identified the major villages (clusters) that are served by the study hospitals. Bwindi hospital serves mainly the sub-counties of Mpungu and Kayonza, while Kisiizi hospital serves mainly Nyarushanje sub-county. All villages in the major catchment areas were listed; there were 183 villages in Nyarushanje sub-county for Kisiizi hospital, and 152 villages (18 for Mpungu sub-county and 134 for Kayonza sub-county) for Bwindi hospital. For each of the two catchment areas, computer-generated random numbers were assigned to all the villages (clusters) and the first 60 villages were selected for the study.

Second, in each of the selected villages, with the help of the village health teams, the survey team listed all households with a child 0–12 months old during the survey period. If the number of eligible households in a village was more than 15, simple random sampling using computer-generated random numbers was applied to recruit the target number of households. If the number of eligible households was less than 15, a nearby cluster was then annexed. If a household had more than one eligible woman, the one with the youngest child was recruited and interviewed in order to reduce recall bias.

### Study variables

The primary dependent variable was health facility delivery service utilization (whether mother sought skilled birth attendance or not). For those who delivered in a health facility, the secondary dependent variable was utilization of a private over a public health facility for child delivery. The explanatory variables included socio-demographic characteristics (Age of mother, parity, education level, marital status, occupation, wealth index), individual perceptions of the importance of skilled health workers, caesarean services, perceived quality of WASH services and quality of care. The wealth quintiles were generated using principal component analysis based on the information collected on assets owned and household structure.

### Data collection and management

A semi-structured questionnaire was developed in English and translated to *Lunyakitara*, the local language of the study area. The *Lunyakitara* questionnaire was back-translated to English, and both English copies were compared for consistency. The English questionnaire, which is provided as supplementary file 1, was developed after reviewing relevant literature [9, 16, 17, 30]. Trained research assistants interviewed mothers using the local language paper questionnaire but recorded the responses on the English questionnaires. The questionnaire captured de-identifiable data on the mothers’ socio-demographics, awareness of the services at the health facility, antenatal visits, distance to the nearest health facility, place of delivery of the most recent child, reasons for utilizing delivery place, as well as challenges experienced while at the health facility. For quality control, all questionable data were reviewed and resolved by a member of the supervisory team every day.

### Data entry and analysis

Descriptive statistics, such as frequencies and proportions, were used to summarize categorical data, while continuous data was expressed as means and standard deviations. To assess the determinants of utilization of a health facility for childbirth, the outcome (delivery place) was dichotomized into health facility delivery coded as 1 or non-facility delivery coded as 0. Any delivery that occurred outside the health facility including at home was considered as a non-facility delivery. We ran a generalized linear model of the Poisson family with logarithm as the link function with robust standard error variances to obtain the prevalence ratios and their 95% confidence intervals for the factors associated with health facility births. Prevalence ratios were used instead of odds ratios since odds ratios tend to overestimate effect of predictor variables when probability of obtaining the outcome of interest is > 10% [31]. To assess the factors associated with utilization of public versus private facility for child delivery, a similar regression technique was used. In both cases, covariates that had a *p* value ≤0.1 in bivariate analysis and those with biological plausibility were considered in the final model building. Stepwise backward elimination method was employed to generate a parsimonious model from a saturated model. Variables were removed stepwise from the model starting with those with highest *p* values until only those variables with *p* values ≤0.05 and/or that significantly improved model fitness were retained. The adjusted prevalence ratios (PRs), their 95% confidence intervals and *p* values are presented. All analyses were performed using STATA 14.0 (StataCorp, Texas,USA).

## Results

A total of 894 mothers of mean age 26.6 years (SD = 5.97) participated in the study representing response rate of 99.3%. The majority of the mothers were married (87.8%) and peasants (74.5%). More than half had 1–2 children (55.4%) and had primary school as their highest level of education (55.0%). Less than half of the participants (46.6%) had resided in the area for 5 years or less (Table 2).

**Table 2:** Socio-demographic characteristics of participants

Variables	Frequency (*n* = 894)	Frequency (%)
**Age category (years)**	(Mean = 26.6, SD = ±5.97)	
16–25	447	50.0
26–35	370	41.4
36–45	77	8.6
**Marital status**		
Single	67	7.5
Married	785	87.8
Separated/widowed/divorced	42	4.7
**Highest level of education**		
None	69	7.7
Primary	492	55.0
Secondary	252	28.2
Tertiary	81	9.1
**Occupation**		
Peasant farmers	666	74.5
Small business	128	14.3
Employed	100	11.2
**Parity (Number of children)**		
1–2	495	55.4
3–4	259	29.0
5 and above	140	15.7
**Length of stay in the area (years)**	(Mean = 9.67, SD = ±9.56)	
0–5	417	46.6
6–10	197	22.0
> 10	280	31.3
**Monthly household income (Uganda shillings)** (Mean ± SD)	(130, 839.5 ± 199,369.9)	
**Wealth quintile**		
Lowest	179	20.0
Second	181	20.3
Middle	188	21.0
Fourth	169	18.9
Highest	177	19.8

### Health facility delivery service utilization

A total of 806 mothers (90.2%) delivered their most recent child in a health facility**.** Of the 806 mothers who delivered in health facilities, 85.6% delivered in a private health facility while 14.3% delivered in public facilities.

Of the 88 (9.8%) women who did not deliver from the health facilities, 68.2% (60) delivered at home, 8.0% (7) at the home of a traditional birth attendant, and 23.9% (21) of the deliveries occurred elsewhere, including on the way to the health facility. The most common reasons for non-facility deliveries included faster progression of labor 77.3% (68), long distance to the facility 25.0% (22), no transport means available 31.8% (28), higher cost of hospital delivery 12.5% (11), lack of funds for transport 18.2% (16) and 5.7% (5) who believed it was not necessary to deliver at a health facility.

### Factors associated with health facility delivery service utilization

In this study, women who were engaged in business as an occupation were 6% more likely to deliver from health facilities compared to peasants [APR = 1.06, 95% CI (1.01–1.11)]. Women with parity of 3–4 [APR = 0.93, 95% CI (0.88–0.99)] were 7% less likely to deliver in health facilities compared to those with parity of 1–2. Women belonging to the highest wealth quintile [APR = 1.09, 95% CI (1.02–1.17)] were 9% more likely to have health based deliveries as compared to those in the lowest quintile (Table 3).

**Table 3:** Factors associated with health facility delivery among women in Kanungu and Rukungiri districts

Variables	Place of delivery		Crude PR (95% CI)	Adjusted PRs (95% CIs)	*P* value
	Home (***N*** = 88)	health facility (***N*** = 806)			
**District**					
Rukungiri	26 (5.7)	427 (94.3)	1	1	
Kanungu	62 (14.1)	379 (85.9)	1.10 (1.05–1.15)*	1.10 (1.05–1.15)*	**<** 0.001
**Age of mother**					
16–25	35 (7.8)	412 (92.2)	1	1	
26–35	39 (10.5)	331 (89.5)	0.97 (0.93–1.01)	1.00 (0.94–1.06)	0.972
36–45	14 (18.2)	63 (81.8)	0.89 (080–0.99)*	0.94 (0.83–1.07)	0.348
**Education status of mothers**					
None	9 (13.0)	60 (87.0)	0.99 (0.90–1.10)		
Primary	62 (12.6)	430 (87.4)	1		
Secondary	14 (5.6)	238 (94.4)	1.08 (1.03–1.13)*		
Tertiary	3 (3.7)	78 (96.3)	1.10 (1.04–1.16)*		
**Marital status**					
Married or cohabiting	74 (9.4)	711 (90.6)	1		
Single	10 (14.9)	57 (85.1)	0.94 (0.85–1.04)		
Divorced/separated	04 (9.5)	38 (90.5)	1.00 (0.90–1.10)		
**Occupation**					
Peasant farmers	79 (11.9)	587 (88.1)	1	1	
Business person	05 (3.9)	123 (96.1)	1.09 (1.04–1.14)*	1.06 (1.01–1.11)*	0.023
Employed	4 (‘4.0)	96 (96.0)	1.09 (1.03–1.14)*	1.05 (0.99–1.10)^+^	0.080
**Parity**					
1–2	31 (6.3)	464 (93.7)	1	1	
3–4	34 (13.1)	225 (86.9)	0.93 (0.88–0.98)*	0.93 (0.88–0.99)*	0.046
≥ 5	23 (16.4)	117 (83.6)	0.89 (0.83–0.96)*	0.93 (0.85–1.02)	0.156
**Distance to the nearest facility (Km)**					
< 5	56 (8.6)	593 (91.4)	1		
≥ 5	32 (13.1)	213 (86.9)	0.95 (0.90–1.00)^+^		
**Wealth quintile**					
Lowest	27 (15.1)	152 (84.9)	1	1	
Second	17 (9.4)	164 (90.6)	1.06 (0.99–1.15)	1.06 (0.98–1.14)	0.131
Middle	17 (9.0)	171 (91.0)	1.07 (0.99–1.16)^+^	1.06 (0.98–1.14)	0.128
Fourth	19 (11.2)	150 (95.5)	1.05 (0.96–1.13)	1.04 (0.95–1.12)	0.402
Highest	08 (4.5)	169 (95.5)	1.12 (1.05–1.21)*	1.09 (1.02–1.17)	0.012
**Attended ANC**					
No	14 (15.9)	74 (84.1)	1		
Yes	74 (9.2)	732 (90.8)	1.08 (0.98–1.19)		

*ANC* Antenatal Care, *PR* Prevalence Ratio; *P values ≤0.05, ^+^ marginal significance (*p* value < 0.1). At multivariate analysis, we adjusted for age, and education status of the mother

### Factors associated with utilisation of private or public health facilities for childbirth

Mothers who valued high skilled health workers during child delivery had a 13% higher likelihood of delivering in a private health facility over a public health facility [APR = 1.13, 95% CI (1.03–1.24)]. Mothers who valued higher quality WASH services were more likely to deliver in private facilities than public facilities [APR = 1.11, 95% CI (1.04–1.18)]. Those who considered the cost of delivery as important when choosing a health facility to deliver in were 15% less likely to deliver in a private facility [APR = 0.85, 95% CI (0.78–0.92)]. Those who considered the presence of caesarean services as important were more likely to choose a private health facility over a public facility [APR = 1.13, 95% CI (1.08–1.19)] (Table 4).

**Table 4:** Factors associated with utilization of private over public health facilities for child delivery

Variables	Choice of facility for child delivery		Crude PR (95% CI)	Adjusted PRs (95% CIs)	*P* Value
	Public (*N* = 116)	Private (*N* = 690)			
**Age of mother**					
16–25	67 (16.3)	345 (83.7)	1		
26–35	41 (12.4)	290 (87.6)	1.05 (0.99–1.11)		
36–45	8 (12.7)	55 (87.3)	1.04 (0.94–1.16)		
**Education status of mothers**					
None	6 (10.0)	54 (90.0)	1.08 (0.98–1.18)		
Primary	71 (16.5)	359 (83.5)	1		
Secondary	29 (12.2)	209 (87.8)	1.05 (0.99–1.12)		
Tertiary	10 (12.8)	68 (87.2)	1.04 (0.95–1.15)		
**Marital status**					
Married or cohabiting	95 (13.4)	616 (86.6)	1	1	
Single / unmarried	11 (19.3)	46 (80.7)	0.93 (0.82–1.06)	0.91 (0.81–1.03)	0.144
Divorced/separated	10 (26.3)	28 (73.6)	0.85 (0.70–1.03)^+^	0.87 (0.72–1.06)	0.161
**Occupation**					
Peasant farmers	89 (15.2)	498 (84.8)	1		
Business person	14 (11.4)	109 (88.6)	1.04 (0.97–1.12)		
Employed	13 (13.5)	83 (86.5)	1.02 (0.93–1.11)		
**Parity**					
1–2	68 (14.7)	396 (85.3)	1		
3–4	30 (13.3)	195 (86.7)	1.01 (0.95–1.08)		
≥ 5	18 (15.4)	99 (84.6)	0.99 (0.91–1.08)		
**Wealth quintile**					
Lowest	22 (14.5)	130 (85.5)	1		
Second	26 (15.9)	138 (84.2)	0.98 (0.89–1.08)		
Middle	30 (17.5)	141 (82.5)	0.96 (0.87–1.06)		
Fourth	16 (10.7)	134 (82.5)	1.04 (0.96–1.14)		
Highest	22 (13.0)	147 (87.0)	1.02 (0.93–1.11)		
**Short distance to the health facility**					
No	57 (11.2)	453(88.8)	1	1	
Yes	59 (19.9)	237 (80.1)	0.90 (0.84–0.96)*	0.92 (0.86–0.98)	0.008
**Skilled health workers available**					
No	54 (25.1)	161 (74.9)	1	1	
Yes	62 (10.5)	529 (89.5)	1.19 (1.10–1.30)*	1.13 (1.03–1.24)*	0.013
**Perceived quality of WASH services at facility**					
Poor	93 (20.3)	366 (79.7)	1	1	
Good	23 (6.6)	324 (93.4)	1.17 (1.11–1.24)*	1.11 (1.04–1.18)*	0.002
**Affordable cost of services**					
No	70 (11.4)	542 (88.6)	1	1	
Yes	46 (23.7)	148 (76.3)	0.86 (0.79–0.94)*	0.85 (0.78–0.92)*	< 0.001
**Availability of medicines**					
Unreliable	84 (19.9)	338 (80.1)	1	1	
Reliable	32 (8.3)	352 (91.7)	1.14 (1.08–1.21)*	1.02 (0.95–1.09)	0.580
**Presence of theatre / caesarean services**					
No	112 (16.9)	549 (83.1)	1	1	
Yes	4 (2.8)	141 (97.2)	1.17 (1.12–1.22)*	1.13 (1.08–1.19)*	0.001

*CI* Confidence Interval, *PR* Prevalence Ratio. At multivariate analysis, we adjusted for age, and education status of the mother. *P values ≤0.05, + marginal significance (*p* value < 0.1)

## Discussion

This study used a sample of mothers who had delivered in the last 1 year, to establish the factors associated with health facility births, and draw comparisons between private and public health facilities. Overall, 90.2% of the mothers had delivered their most recent child in a health facility; with most delivering in private facilities rather than in public facilities. Although high health facility delivery utilization has been reported in studies in Kenya [32, 33], findings from other studies in Africa and South Asia have found relatively low proportions of health facility deliveries [34–37]. The higher proportion of hospital deliveries in the current study can be attributed to the fact that our study was done within the catchment of major hospitals in the two study districts. Another explanation for high hospital deliveries is the community health insurance scheme that was implemented at the two private hospitals where mothers who attended antenatal clinics at those hospitals were given subsidized services. This could have encouraged more mothers to deliver in those health facilities. A relatively high proportion of mothers delivered in private facilities rather than public facilities, in line with a study in Nairobi, Kenya [32] but not consistent with another study in Ethiopia [38]. In our study, private hospitals were better equipped and staffed to handle obstetric complications. We can also attribute this preference to the general perception that private facilities provide better quality care and can better handle obstetric complications as highlighted in other studies [39, 40]. On the other hand, the reasons identified for opting for non-facility deliveries included sudden onset of labour, high costs of hospital deliveries, poor access to transportation, and long distance to facilities, which have been, previously reported [41–44].

We found a significant association between maternal occupation (being a business-woman) and facility delivery service utilization which is similar to findings of a study conducted in Ethiopia [45]. This finding is understandable because these business-women can afford the cost of hospital care and therefore tend to opt for hospital deliveries, unlike the women peasant farmers. We also found a significant positive association between being in the highest wealth quintile and facility delivery utilization. Prior studies have also shown an association between higher wealth quintile of the mothers and utilization of facility delivery services [46, 47]. A study in South Asia indicated that the proportion of private facility deliveries increased with the level of wealth [34], implying that increased socioeconomic index enhances the likelihood of health facility delivery service utilization.

Women with high parities (parity of 3–4) were less likely to opt for facility deliveries. This is consistent with previous studies [48–50]. One of the possible reasons for the low utilization of health facility delivery among multiparous women could be that they have prior experience and knowledge from previous pregnancies and deliveries and feel more confident that they can deliver safely and hence facility delivery is a lower priority. A study by Alemi suggests that women with low parity could be more motivated to deliver in health facilities due to fear of labour complications compared to their counterparts, and usually they are accompanied by relatives or husbands for their first delivery [50].

Among mothers who utilized health facilities, delivery in private health facilities was significantly associated with mothers’ perceptions of availability of skilled health workforce, perceived WASH conditions of the facility, perceived cost of delivery services, and availability of caesarean services. The provision of quality WASH services in health care facilities is paramount to ensure safety, comfort and privacy of the mothers. In this study, mothers who valued higher quality WASH services were less likely to deliver at public health facilities when compared to private facilities. However, it is important to note that this study was conducted in the catchment area of two major private health facilities with active WASH projects. Though the effect of WASH in health facilities on health seeking behaviour has not been extensively studied, improved WASH conditions, such as continuous supply of improved water, and provision of improved toilets, bathing facilities and hand washing facilities, are likely to contribute to a better childbirth experience and reduce the risk of healthcare-associated infections among mothers and new-borns [51].

The availability of caesarean services also attracted mothers to deliver at private facilities compared to public facilities. Perceptions that private facilities provide better quality services during obstetric complications have been reported [39]. Public health facilities have been reported to lack some essential services, including caesarean services, and at times are overwhelmed by demand. Lack of surgical theatres and the limited number of trained staff are often identified with public facilities, and this can discourage mothers from choosing to deliver at public facilities in favour of private ones [52, 53]. Private facilities in our study also had a relatively higher number of skilled health workers than in the neighbouring government facilities. Having more healthcare providers in private facilities may mean more responsiveness to patients’ needs and shorter waiting times for patients as highlighted in the literature [54]. It is likely that similar reasons related to the adequacy of the healthcare workforce, responsiveness, and waiting time could have affected mothers’ utilization of health facilities for childbirth in our study though these were not specifically explored by the survey.

The cost of health facility delivery was also associated with the type of health facility utilized for child delivery. Mothers were less likely to report delivering in private health facilities compared to public facilities due to the higher cost of delivery. Private facilities have been known to be relatively more expensive than government facilities for most services [55, 56], and therefore many mothers without health insurance usually face challenges in accessing services in such facilities. The cost of services can therefore discourage mothers who cannot afford the bills from private health facilities.

### Limitations

In a cross-sectional study, our main limitation is that we cannot infer causal relationships. Secondly, the responses of mothers were self-reported, and as such response and courtesy bias may have affected the participants’ responses; i.e., perceived social pressure to respond to the questions in a certain way. However, the interviews were conducted by well-trained research assistants who objectively interviewed the mothers. The third limitation, is that the study included some mothers whose children were 12 months old, thus there is a possibility of recall bias because some women may not have been able to remember all the circumstances related to a child delivery that happened 1 year prior to the survey. Besides, the study did not address respectful maternal care, and respondents were selected from catchment areas of private and public health facilities. Selection of respondents from catchment areas of private and public health facilities may provide results, which may not reflect the health-seeking behavior for mothers living in rural communities with limited access to health facilities. However, this is one of the few studies on factors associated with health facility deliveries in rural Uganda, and it provides critical comparisons between private and public health facilities, hence adding to the growing body of evidence of the complex issues affecting maternal health service utilization.

## Conclusion

In these study areas, there was high utilization of health facilities for childbirth. Wealth index, maternal occupation, and parity were significant determinants of health facility delivery. More deliveries occurred in private health facilities than in public health facilities. Utilization of private versus public facility to deliver a child was associated with the perceived quality of WASH services at the facility, availability of skilled birth attendants, cost of delivery services, and availability of caesarean services. It is important to improve WASH conditions and capacity for providing caesarean deliveries as well as recruit more skilled birth attendants in all health facilities. Non-facility deliveries were related to faster progression of labour, high costs of facility delivery, and transport challenges, which should be addressed to ensure that more mothers are able to deliver in health facilities and thereby reduce maternal mortality.

This is one of the few studies that have assessed factors associated with health facility deliveries among mothers in rural Uganda. Further, our community-based study was the first of its kind in rural Uganda, to reveal that perceived quality of WASH services is associated with mothers’ utilization of private over public facilities for childbirth. More research to explore mechanisms of improving WASH services delivery and sustainability in health facilities, and mothers’ perspectives and experiences on WASH in health facilities is recommended.

## Supplementary Information

**Additional file 1.**

## Data Availability

The datasets analysed during the current study are available from the corresponding author on reasonable request.
