# Supplementary material for: Factors associated with health facility deliveries among mothers living in hospital catchment areas in Rukungiri and Kanungu districts, Uganda
Source: BMC Pregnancy Childbirth. 2021 Apr 26;21:329. doi: 10.1186/s12884-021-03789-3 (PMC8077901; doi:10.1186/s12884-021-03789-3)
Supplement: Supplementary file 1 — Additional file 1. [file 12884_2021_3789_MOESM1_ESM.docx]

**FACTORS ASSOCIATED WITH HEALTH FACILITY DELIVERIES AMONG MOTHERS LIVING IN HOSPITAL CATCHMENT AREAS IN RUKUNGIRI AND KANUNGU DISTRICTS, UGANDA.**

**Household questionnaire.**

Richard K. Mugambe^1*^, Habib Yakubu^2^, Solomon Tsebeni Wafula^1^, Tonny Ssekamatte^1^, Simon Kasasa^3^, John Bosco Isunju^1^, Abdullah Ali Halage^1^, Jimmy Osuret^1^, Constance Bwire^1^, John C. Ssempebwa^1^, Yuke Wang^2^, Joanne A. McGriff^2^, Christine L. Moe^2^.

1. Department of Disease Control and Environmental Health, School of Public Health, Kampala, College of Health Sciences, Makerere University, P.O Box 7072, Kampala, Uganda.
2. The Center for Global Safe Water, Sanitation and Hygiene. Rollins School of Public Health, Emory University, 1518 Clifton Rd. NE, Atlanta, GA 30322, USA.
3. Department of Epidemiology and Biostatistics, School of Public Health, Kampala, College of Health Sciences, Makerere University, P.O Box 7072, Kampala, Uganda.

**PART I: GENERAL INFORMATION**

| **No.** | **Prompt** | **Response** |
| --- | --- | --- |
| 1.1 | Date of interview (DD/MM/YY) | \|___\|___\|2018 |
| 1.2 | Questionnaire number | \|__\|__\|__\| |
| 1.3 | Parish |  |
| 1.4 | Village |  |
| 1.5 | District | 1. Kanungu 2. Rukungiri |
| 1.6 | Start time |  |
| 1.7 | Name of interviewer |  |

**PART II: SOCIO-DEMOGRAPHIC CHARACTERISTICS OF MOTHERS**

| **No.** | | **Prompt** | **Response *(please circle)*** |
| --- | --- | --- | --- |
|  | | How old are you? (Record age in completed years) | ___________years |
|  | | What is your religion? | 1= Catholic  2= Anglican  3= Muslim  4= Pentecostal/ Born again  5= Seventh Day Adventists (SDA)  6= Other (specify) ________________ |
|  | | What is the highest level of education you attained? | 1= None  2= Primary (P1 to P7)  3= Secondary  4= Tertiary |
|  | | Parity [Number births] | _____________ |
|  | | Number of children alive |  |
|  | | What is your marital status? | 1= Single  2= Married/cohabiting  3= Widowed  4= Divorced/separated |
|  | | What is your occupation? | 1=Peasant  2=Casual labourer  3=Business person  4=Salaried worker  5=Unemployed  6=Other (specify) _____________ |
|  | | How long have you lived in this village? (years) | ________ (months)  ________ (years) |
|  | | What do you mainly use for lighting the house you live in? | 1= Electricity  2= Solar  3= Kerosene lamp  4= Kerosene candle  5= Wax candle  6= Other (specify) |
| **Wealth Index** | | | |
|  | What is the average monthly income of the HH head? (Uganda Shillings) | | _____________Uganda shillings |
| **A) ASSET OWNERSHIP:** Does your household have? (The asset has to be **FUNCTIONAL**) | | | |
|  | | A radio | 1=YES 2=NO |
|  | | A mobile phone | 1=YES 2=NO |
|  | | A Television | 1=YES 2=NO |
|  | | A Motorcycle | 1=YES 2=NO |
|  | | A Car | 1=YES 2=NO |
|  | | A Bicycle | 1=YES 2=NO |
|  | | A manufactured bed | 1=YES 2=NO |
|  | | Apiece of land | 1=YES 2=NO |
|  | | Large farm animals like cattle, goats and sheep | 1=YES 2=NO |
|  | | Small farm animals like poultry | 1=YES 2=NO |
|  | | Walls of main dwelling house permanent | 1=YES 2=NO |
| **DWELLING UNIT CONSTRUCTION AND CHARACTERISTICS (OBSERVE)** | | | |
|  | | B1=FLOOR MATERIAL | 1=Earth  2= Earth And Dung  3=Parquet or Polished Wood  4= Mosaic or Tiles  5= Bricks  6=Cement  7=Stones  8=Concrete  9=Other (Specify)_____________ |
|  | | B2=WALL MATERIAL | 1**=**Thatch/Straw  2=Mud and Poles  3=Un-Burnt Bricks  4=Un-Burnt Bricks with Plaster 5=Burnt Bricks with Mud  6=Cement Blocks  7= Stone  8= Timber  9=Burnt Bricks with Cement  10=Metal/ iron sheets  11= Other (Specify) |
|  | | B3=ROOF MATERIAL | 1=Thatch  2= Iron Sheets  3= Tiles  4=Others (Specify) _____________ |
| **ACCESS TO RESOURCES** | | | |
| **C1:** **DRINKING WATER** | | | |
|  | | What is the main source of drinking water for members of your household? | 1=Public taps/ stand pipes  2=Boreholes  3=Protected dug well  4=Unprotected dug well  5=Protected spring  6=Unprotected spring  7=Surface water (dams, lakes ,rivers, stream, ponds and canal  8=Covered rain water tank  9=Uncovered rain water tank  10=Cart with tank  11=Piped household water connection located inside the house, plot or yard  12=Other |
|  | | How long does it take to go to the water source, get water, and come back? (on foot) | _____________in minutes |
| **C2: SANITATION** | | | |
|  | | Does your household have a latrine facility? | 1=Yes  2=No (if No, go skip next question) |
|  | | If yes, what type of sanitation facility do you use?  (If respondent has answered this, skip next question) | 1=Flush toilet  2= VIP Latrine  3= Traditional pit latrine  4= Shared private  5= Public facility  6=Ecosan  7= Other (specify) |
|  | | If No, what do you use? | 1= Shared public  2=Shared private  3=Open defecation/ Bush |
| **C3: ENERGY** | | | |
|  | | What type of fuel does your household mainly use for cooking? (Tick only one response) | 1= Firewood  2= Charcoal/ briquettes  4=Gas  5= Biogas  6= Electricity  7=Kerosene/Paraffin  8=Straw/Shrubs/Grass 9=Animal Dung  10=No Food Cooked In Household 11= Others (Specify)_____________ |
|  | | Do you have a separate room which is used as a kitchen? | **1=**Yes 2=No |

**PART III: SEEKING OF MCH SERVICES**

|  | Did you ever seek MCH services during your most recent pregnancy from a health facility in this area? If No, go to 3.4. | 1=Yes 2=No |
| --- | --- | --- |
|  | If Yes, which type of health facility did you seek MCH services from? | 1=Public facility [name] _____________  2=PNFP facility [name] _____________  3=Private facility [name] _____________ |
|  | Which MCH services did you seek from the nearby health facility during the most recent pregnancy? | \| ANC \| 1=Yes  2=No \| \| --- \| --- \| \| Delivery services \| 1=Yes  2=No \| \| Post-natal care \| 1=Yes  2=No \| \| Neonatal care \| 1=Yes  2=No \| \| Others (specify) \| 1=Yes  2=No \| |
|  | Where did you deliver your youngest child?  If delivery was not at a health facility skip to 3.15 | 1= Public Facility [name] _____________  2=PNFP facility [name] _____________  3=Private facility _____________  4=Traditional Birth Attendants (TBA)  5=At home  6=Others e.g. on the way to health facility (specify)_____________ |
|  | In 3.4 above, if delivery was in a health facility, what was the main reason for delivering your youngest child from the above facility? (Circle only one reason) | 1=Short distance to the health facility  2=Availability of skilled health workers  3=Availability of medicines all the time  4=Good WASH services  5=Availability of adequate lighting  6=Availability of a caesarian section  7=Affordable cost of services  8=Others (specify)_____________ |
|  | In 3.4 above, if delivery was in a health facility, what were the other reasons for delivering your youngest child from the above facility? (Multiple choice responses) | 1=Short distance to the health facility  2=Availability of skilled health workers  3=Availability of medicines  4=Good WASH services  5=Affordable cost of services  6=Others (specify)_____________ |
|  | In case you are to deliver another child, would you deliver at the same health facility where you delivered your youngest child? | 1= Yes  2= No |
|  | If yes in 3.7 above, why? | 1=Short distance to the health facility  2=Availability of skilled health workers  3=Availability of medicines all the time  4=Good WASH services  5=Affordable cost of services  6=Others (specify) _____________ |
|  | If no in 3.7 above, why? | 1=Long distance to the health facility  2=Lack of skilled health workers  3=Lack of medicines all the time  4=Poor WASH services  5=High cost of services  6=Others (specify) _____________ |
|  | Would you recommend other mothers to seek delivery services from the facility where you delivered your youngest child? | 1=Yes  2=No |
|  | If yes in 3.10 above, why? | 1=Short distance to the health facility  2=Availability of skilled health workers  3=Availability of medicines all the time  4=Good WASH services  5=Affordable cost of services  6=Others (specify) _____________ |
|  | If no in 3.10 above, why? | 1=Long distance to the health facility  2=Lack of skilled health workers  3=Lack of medicines all the time  4=Poor WASH services  5=High cost of services  6=Others (specify _____________ |
|  | In 3.4 above, if delivery was in a health facility, what was the water, sanitation and hygiene status in the health facility where you delivered your youngest child? | \| Separate latrines for men and women \| 1=Yes \| 2=No \| \| --- \| --- \| --- \| \| Hand washing facilities outside the latrine \| 1=Yes \| 2=No \| \| Presence of water and soap for hand washing outside latrine \| 1=Yes \| 2=No \| \| Carried water from home to the health facility on the day of delivery \| 1=Yes \| 2=No \| \| Clean walls and floors in health facility \| 1=Yes \| 2=No \| \| Latrines with pit covers \| 1=Yes \| 2=No \| |
|  | In 3.4 above, if delivery wasn’t at a health facility, what was the main reason for not delivering your youngest child from the health facility? (Circle only one reason) | 1= Poor WASH conditions at the health facility  2=Formal cost of treatment too high  3=Informal cost of treatment too much  4=Facility not open  5=Facility too far  6=No transport (Vehicle, boda boda, bicycle) available  7=No money for transport  8=Labour progressed too fast  9=Do not trust facility/poor quality of service  10=No female provider at the facility  11=Husband family did not allow  12=Not necessary to deliver in a H/facility  13=No provider at the facility  14=Did not have required supplies (gloves, mackintosh etc.)  15=TBA’s accessibility  16=Others specify |
|  | In 3.4 above, if delivery wasn’t at a health facility, what were the other reasons for not delivering your youngest child from a health facility? (Refer to health facility at 3.4) (Multiple choice responses) | 1= Poor WASH conditions at the health facility  2=Formal cost of treatment too much  3=Informal cost of treatment too much  4=Facility not open  5=Facility too far  6=No transport (Vehicle, boda boda, bicycle) available  7=No money for transport  8=Labour progressed too fast  9=Do not trust facility/poor quality of service  10=No female provider at the facility  11=Husband family did not allow  12=Not necessary to deliver in a H/facility  13=No provider at the facility  14=Did not have required supplies (gloves, mackintosh etc.)  15=TBA’s accessibility  16=Others specify |
|  | In case you are to deliver another child, would you make a change from Home/TBA to a health facility? | 1= Yes  2= No |
|  | If yes in 3.16 above, give reasons. | …………………………………………………………………………………………………………………………………………………………………………………………………………………………………………….. |
| **PART IV:**  **CHALLENGES RELATED TO ACCESSING DELIVERY SERVICES** | | |
|  | Did you make any payments towards delivery services at place where you delivered your recent child? | 1= Yes  2= No |
| 4.2 | If yes, specify how much and for what? | _____________ |
| 4.3 | What challenges did you experience when you delivered your recent child? | 1=Poor WASH services  2=Unskilled birth attendants  3=Long waiting in long lines  4=Poor health-worker attitude  5=Expensive services  6=Others (specify) _____________ |
| 4.4 | Distance to place where you delivered your recent child? | [Km] _____________ |
| 4.5 | What type of transport means did you use to get to the place where you delivered your recent child? (If respondent walks, skip 4.7). | 1=Walking  2=Bicycle  3=Motorcycle  4=Car  5=Other (specify) |
| 4.6 | How much do you pay to use the above transport means? |  |
| 4.7 | How long does it take to walk to the place where you delivered your youngest/recent child? | 1=Less than 30 minutes  2=Between 30 min- 1hr  3=Between 1hr-2hrs  4=More than 2hrs |
| 4.8 | To what extent were you satisfied with the delivery services at the place where you delivered? | 1=Very satisfied  2=Satisfied  3=Neutral  4=Dissatisfied  5=Very dissatisfied  6=Not Applicable (Didn’t deliver at health facility) |
| 4.9 | If satisfied in 4.8 above, what are the reasons for your satisfaction? | 1=Availability of water  2=Availability of clean latrines  3=Clean environment  4=Availability of hand washing facilities  5=Availability of utilities (electricity etc.,)  6=Skilled birth attendants  7=Short/no waiting in long lines  8=Good health-worker attitude  9=Affordable services  10=Availability of medicines and supplies  12=Others (Specify) _____________ |
| 4.10 | If dissatisfied in 4.8 above, what are the reasons for your dissatisfaction? | 1=Lack of water  2= Lack of clean latrines  3= Dirty environment  4= Lack of hand washing facilities  5= Lack of utilities (electricity etc.,)  6= Lack of skilled birth attendants  7=Long waiting in long lines  8=Poor health-worker attitude  9=Expensive services  10= Lack of medicines and supplies  12=Others (Specify) _____________ |
| 4.11 | What was the main source of water at the place where you delivered your recent child? | 1=Public taps/ stand pipes  2=Boreholes  3=Protected dug well  4=Unprotected dug well  5=Protected spring  6=Unprotected spring  7=Surface water (dams, lakes ,rivers, stream, ponds and canal)  8=Rain water tank  9=Cart with tank / water vendor  10=Piped health facility water connection located inside the house, plot or yard  11=Don’t know  12=Other (specify) |
| 4.12 | Did you make any financial payments towards water services at the place where you delivered your recent child? | 1=Yes  2=No |
| 4.13 | If yes in 4.12 above, specify how much | _____________Quantity_____________ |

**END TIME:** _____________
